# Safranal-promoted differentiation and survival of dopaminergic neurons in an animal model of Parkinson’s disease

**DOI:** 10.1080/13880209.2018.1501705

**Published:** 2018-10-24

**Authors:** Yi Zhao, Gangming Xi

**Affiliations:** Neurology Department of Xuhui Central Hospital, ShangHai, China

**Keywords:** Neural stem cell, tyrosine hydroxylase

## Abstract

**Context:** Safranal (SAF) is verified to have potential effects in promoting nerve growth.

**Objectives**: This study verifies the role of SAF in promoting dopaminergic neurons growth *in vitro* and *in vivo*.

**Material and methods:** Rat neural stem cells (NSC) were treated with 1, 20, or 100 ng/mL of SAF, and the expression levels of tyrosine hydroxylase (TH) and dopamine transporter (DAT) were assayed by flow cytometry and real-time PCR and the secretion of dopamine (DA) was assayed by ELISA. Then, 2 × 10^6^ cells of SAF-treated NSC was administrated into PD rat models induced by 6-OHDA. The differentiation and survival of dopaminergic neurons was identified by fluorescence microscope and TH^+^ cells by immunostaining and DA secretion by ELISA at week 2 and week 4, respectively.

**Results:** After being treated with SAF at 20 and 100 ng/mL for 1 week, TH and DAT positive rates increased 1.4- and 1.7-fold (*p* < 0.01, respectively). TH and DAT mRNA also increased 8.05- and 4.41-fold, respectively. And the release of DA statistically increased 1.5-fold (*p* < 0.01). *In vivo*, the number of rotations decreased to 4.33 ± 0.97 rpm (*p* < 0.01) and the survival rates increased to 77.66 ± 7.87% (*p* < 0.05) at week 4 after transplantation of SAF-treated NSC. Moreover, the transplanted cells increased three-fold, TH fluorescence density increased four-fold and DA releases increased 1.4-fold (*p* < 0.01) at week 4 after transplantation.

**Conclusions:** SAF promoted the production of functional DA cells and alleviated PD, which may contribute to a new therapy for PD patients.

## Introduction

Parkinson’s disease (PD) is a chronic neurodegenerative disease occurring in middle-aged and senior people, characterized by slow progress of dopamine (DA) neuron degeneration and death in the midbrain substantia nigra pars compacta (SNc) resulting in the loss of DA nerve endings in the striatum, causing tremors, muscle rigidity, bradykinesia, postural instability, and a series of syndromes (Farzanehfar 2016). Currently, the causes of death of these neurons are not well-understood. However, genetic and environmental factors such as aging, oxidative stress, reactive oxygen species, over-generation, and cell apoptosis may be implicated in the degeneration death process of dopaminergic neuron (Nakamura et al. 2012). Drug treatment and deep-brain stimulation (DBS) can alleviate the patient’s symptoms to a certain extent, but there are some side effects and it cannot fundamentally curb the sustainable death of DA neurons in the brain. A potential therapeutic approach to PD which has been developed for the last decade is the implantation of DA-producing cells into the striatum, which not only replaces part of the lost DA neurons function, but also secretes neurotrophic factors to inhibit the loss of DA neurons. At present, stem cells are widely used for cell-replacement therapy in PD (Hu et al. 2015). However, stem cells are potentially carcinogenic and this is an invasive treatment. So, it is not a reliable widespread therapy to use.

*Crocus sativus* L. (Iridaceae) has been used from ancient times as an herbal medicine. Recently, a growing volume of data has demonstrated that the main component, safranal (SAF), has neuroprotective effects; however, the mechanisms remain unclear. SAF has anti-hyperglycemic and antioxidant properties and thus is useful in preventing or slowing down diabetic peripheral neuropathy (Farshid and Tamaddonfard 2015). SAF has been found to lessen the alloying effect induced by spinal nerve transection (Zhu and Yang 2014). Moreover, a recent study provided evidence that SAF improved histopathology of an injured peripheral nerve (Tamaddonfard et al. 2014). The target and mechanism of SAF in the brain is still unclear though.

In the present study, we seek to prove the effects of SAF on the proliferation and differentiation of DA-producing cells and the use of SAF in the treatment of PD diseases. In order to trace the transplanted cells in the brain for long-term survival, growth, migration, and differentiation processes, we used enhanced green fluorescent protein (EGFP) to label differentiated cells and administrated them into brain of a PD animal model.

## Materials and methods

### Ethics statement

Wild-type Sprague-Dawley (SD) rats (220–250 g) were supplied by Laboratory Animal Center, Xuhui Central Hospital and maintained under specific pathogen-free condition (Animal Center, Xuhui Central Hospital). All experiments referring to the use of animals were approved by the Committee of Animal Care and Use of Xuhui Central Hospital.

### Rat GFP-neural stem cell

Rat GFP-neural stem cell (NSC) was purchased from Cyagen Bioscience, Inc. (Guangzhou, China). The NSC was grown in a SD rat NSC basal medium (Cyagen Bioscience, Inc.) supplemented with 15% SD rat NSC-qualified fetal bovine serum, 1% penicillin–streptomycin, and 1% glutamine at 37 °C in 5% CO_2_ and saturated humidity. The medium was replaced with an additional 1, 20, or 100 ng/mL of SAF (Baomanbio, Shanghai, China) for four weeks prior to assay.

### Flow cytometry assay

Cells from Petri dishes were harvested and washed twice in phosphate-buffered saline (PBS), counted, and re-suspended in 1% BSA containing 0.01% NaN_3_. For flow cytometer analysis, 10^5^ cells were incubated with rabbit monoclonal antibodies to tyrosine hydroxylase (TH) (Abcam, Hangzhou, China) and dopamine transporter (DAT) (Biolegend, San Diego, CA) for 1 h. Then, the cells were washed with washing buffer three times, re-suspended in 0.5 mL PBS, and analyzed by flow cytometry (Beckman Coulter, Atlanta, GA). All incubations were performed on ice. The positive rates were expressed as a percentage of positive cells in the total cells.

### Real-time reverse-transcription polymerase chain reaction

Total RNA was extracted from differentiated cells by using Trizol (Invitrogen, Carlsbad, CA). First-stand cDNA was generated with a Superscript first-stand synthesis kit (Invitrogen, USA). A total of 3 μg RNA treated by DNase was used in each cDNA synthesis, at a total volume of 20 μL. PCR amplification was performed by using SYBR Green Real Time PCR Kit (Bio-Rad, Hercules, CA). The number of cycles was 35 with denaturation at 95 °C for 30 s and elongation at 60 °C for 60 s. The PCR cycle was preceded by an initial denaturation of 5 min at 95 °C and followed by a final extension of 10 min at 72 °C. The primers, used for real-time PCR were 5′-GTTCATCGGACGGCGACAGA-3′ and 5′-TCCCTACCCTTACGACAAGAGT-3′ for TH; 5′-TGGGTTTGGAGTGCTGATTGC-3′ and 5′-GAGGAGACCGAAGCAGCAGAAG-3′ for DAT. GAPDH was used as an internal control (Wang et al. 2015).

### PD models induced by 6-OHDA and transplantation of differentiated cells

SD rats were anesthetized with 60 mg/kg of pentobarbital sodium and injected with 5 μL of 6-hydroxydopamine (6-OHDA, 2 mg/mL, Sigma, St. Louis, MO) below the dura of the right medial forebrain. Four weeks later, the unilaterally 6-OHDA-lesioned rats were tested for rotational behaviour in response to subcutaneous apomorphine (0.5 mg/kg) injections in an automated rotameter as previously described (Toriumi et al. 2009). Animals showing significant ipsilateral rotations (>6 rpm) were used for transplantation studies.

The GFP-NSC was re-suspended in a medium having a concentration of 10^9^ cells/mL. In total, 2 μL of the cell suspensions were used for an intrastriatal injection (Toriumi et al. 2009). To inhibit immunological rejection, injections of cyclosporine A [10 mg/kg, intraperitoneally (i.p.)] were given to the animals daily from 48 h prior to transplantation to 1 week after operation, then followed by the addition of cyclosporine A (0.1 mg/mL) to the drinking water until death. In control animals, 2 μL of medium was injected and the cyclosporine A was given as above. The rats were tested for rotational behavior every week after transplantation. Then, they were anesthetized and perfused transcardially with 4% paraformaldehyde in PBS. The whole brain of each rat was removed and subjected for immunostaining assay.

### Immunostaining on brain slices

Rat brains were perfused overnight and then dehydrated in a series of alcohol gradients (70–100%), embedded in paraffin and sectioned at 6 µm routinely. The specific primary antibodies were rabbit anti-TH (1:500, Abcam) and the secondary antibodies were anti-rabbit Cy3-conjugated antibodies (1:200, Abcam). The immunostaining procedures were performed according to the manufacturer’s instruction and the fluorescence density was quantified by Image J statistic software (Bethesda, MD).

### ELISA assay

ELISA assay (eBioscience, San Diego, CA) was utilized to analyze protein levels of DA from cultured cells or brain tissues according to the manufacturer’s instruction. For cultured cells, 10^6^ cells were made 10% homogenate by adding 0.9 mL PBS, and centrifuged 5000*g* at 4 °C for 15 min. The supernatant (10 μL) was used to determine OD value and the contents were represented by pg/mL. For brain tissues, 1 g of striatum tissues was weighed from the rat’s lesion side, made 10% homogenate by adding 9 mL PBS, and centrifuged 5000*g* at 4 °C for 15 min. The supernatant (10 μL) was used to determine OD value and the contents were represented by pg/mL.

### Statistical analysis

Statistical analysis was performed using the SPSS statistical package (SPSS Inc. Chicago, IL). Differences were evaluated by one-way analysis of variance (ANOVA). *p* value <0.05 was considered statistically significant.

## Results

### Characteristics of TH^+^ cells differentiated from NSC

**Figure 1.**
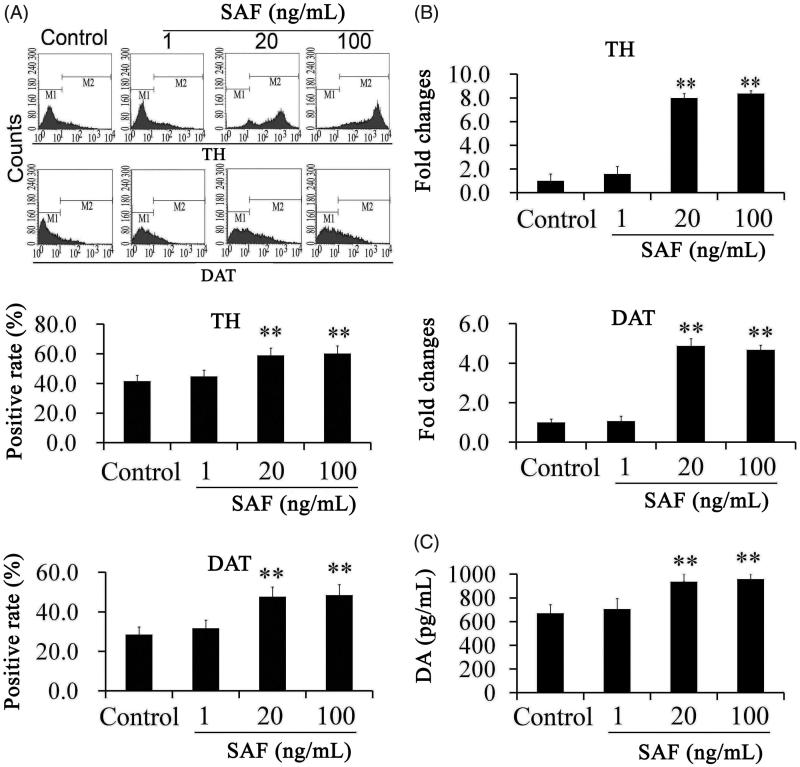
The positive rates of tyrosine hydroxylase (TH) and dopamine transporter (DAT) were assayed by flow cytochemistry (A) and real-time PCR (B) and DA releases were assayed by ELISA (C). TH and DAT positive rates obviously increased in the SAF group at 20 and 100 ng/mL. Moreover, DA releases also increased in the SAF group. **p < 0.01 versus control. The positive rates were expressed as a % of positive cells in the total cells. Differences were evaluated by one-way ANOVA.

After being treated with different concentrations of SAF for 1 week, when compared with the control, there was no significant difference in the typical morphology of neurons. By flow cytometry, we found that the TH positive rate was 41.65 ± 3.14%; and DAT positive rate was 28 ± 2.67% in the control group. When compared with the control group, TH positive rates (55.78 ± 2.38% and 58.44 ± 3.76%, respectively) and DAT positive rates (47.69 ± 3.64% and 48.53 ± 2.87%, respectively) in the SAF group at concentrations of 20 and 100 ng/mL exhibited significant increase. Although, there was a slight increase at the high concentration, there was no significant difference between the two concentrations (Figure 1(A)). SAF had no effects on promoting differentiation of dopaminergic neurons at the low concentration (1 ng/mL).

In addition to the flow cytometry assay, RT-PCR analysis was also performed to further detect three dopaminergic cell markers, including TH and DAT. When compared with the control, TH mRNA increased 7.83- and 8.05-fold, respectively, in the SAF group at 20 and 100 ng/mL; DAT mRNA expression increased 4.38- and 4.41-fold, respectively, in the SAF group at 20 and 100 ng/mL (Figure 1(B)).

ELISA was used to examine the ability of differentiated cells to release DA. The results are shown in Figure 1(C). The release of DA detected in the SAF group at 20 and 100 ng/mL were statistically increased when compared with control group. These observations suggested that the NSC has a dopaminergic neuron function and the SAF promoted the differentiation of dopaminergic neurons.

### Transplantation of GFP-NSC into PD rats

The GFP-NSC were transplanted into the SD rats that had received unilateral lesions with 6-OHDA 4 weeks before and had been tested for anthropomorphic rotation to verify the lesions. Rats showing significant ipsilateral rotations (>6 rpm) after 4 weeks were chosen. The successful rate to make PD models was about 70%. Forty-five rats were used to make PD models, and 30 rats suited for this study were chosen after 4 weeks and divided into three groups: PD group (2 μL of medium), PD + NSC (control group, 2 μL of NSC), and PD + NSC + SAF (100 ng/mL) (SAF group, 2 μL of SAF-treated NSC).

**Figure 2.**
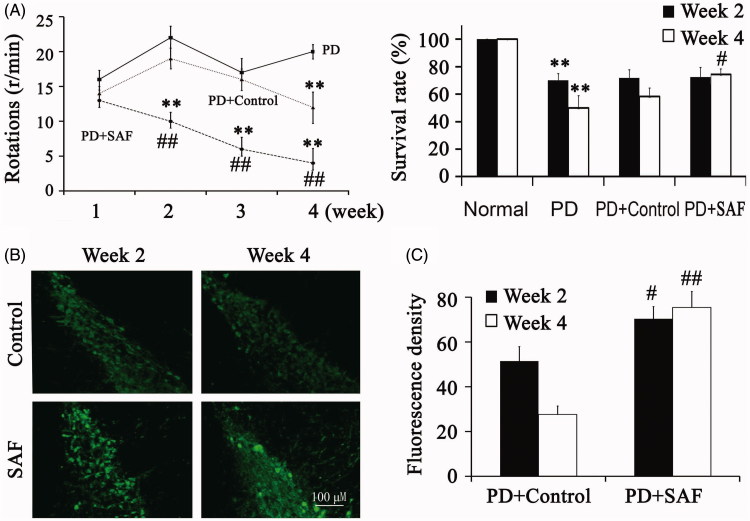
Transplantation of GFP-NSC into PD rats. (A) The number of rotations and the survival rates of animals after transplantation. (B) The distribution and survival of transplanted cells were examined by fluorescence microscopy. **p < 0.01 versus Normal group. ^#^*p* < 0.05 and ^##^*p* < 0.01 versus PD. Differences were evaluated by one-way ANOVA.

The number of rotations in the SAF group after treatment with apomorphine for 30 min decreased significantly from the second week after transplantation, but in the control group, the number of rotations saw a significantly decrease from the fourth week after transplantation (Figure 2(A)). At Week 2 and 4 after transplantation, the survival rates of animals were also shown in Figure 2(A). The SAF group statistically increased at Week 4, when compared with control group.

By using fluorescence microscopy, we found only a small amount of transplanted cells survived in the control group along the injection line. However, more cells survived in the SAF group, which migrated to wider regions from Week 2 (Figure 2(B)).

**Figure 3.**
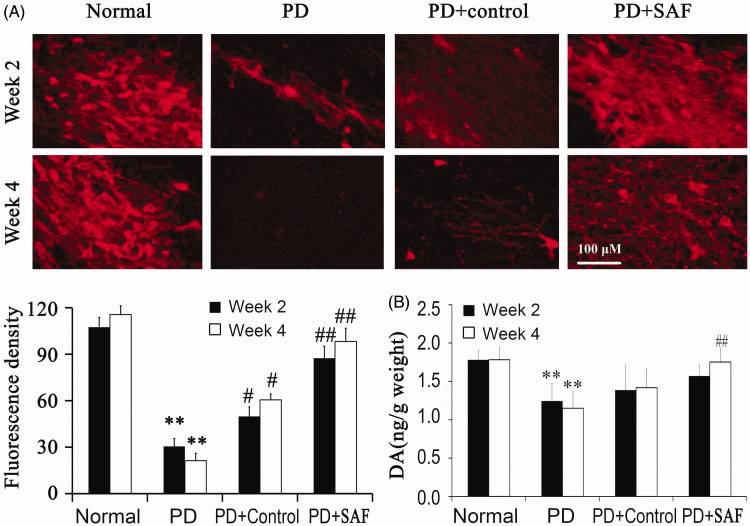
Differentiation and survival of dopaminergic neurons. (A) TH positive rates were examined by immunofluorencence. (B) DA release was examined by ELISA. **p < 0.01 versus Normal group. ^#^*p* < 0.05 and ^##^*p* < 0.01 versus PD. Differences were evaluated by one-way ANOVA.

By immunofluorencence, we found that TH-positive rate decreased obviously in PD group at Week 2 and Week 4. Although NSC alone promoted the differentiation of TH-positive cells, SAF promoted the differentiation of TH-positive cells at Week 2 and 4 when compared with the control group (Figure 3(A)). These observations suggested that SAF promoted the differentiation and survival of dopaminergic neuron function *in vivo*.

DA releases were detected at Week 2 and 4 after transplantation. DA detected in the brain tissues was very low at Week 2 and 4 in PD group and control group; however, it became more obvious at Week 4 in the SAF group (Figure 3(B)).

## Discussion

Oxygen free radicals may be one of the reasons for PD diseases. A series of antioxidants are known to eliminate oxidative damage and are useful in treating PD diseases (Velusamy et al. 2017). Saffron and its active ingredients have been known to treat anxiety, depression and other mental disorders due to their effect in modulating neurotransmitter releases, which are a less toxic and more favorable outcome (Shafiee et al. 2018). SAF also has obvious anti-Parkinson effects including inhibiting a-LA, increasing TH + cells count indicating SAF pretreatment saved many dopaminergic cells in the SNc and retina in MPTP (1-methyl-4-phenyl-1,2,3,6-tetrahydropyridine)-induced PD animal model (Khazdair et al. 2015).

The differentiation of DA neurons from various stem cells has been reported since 2004. At present, coculturing embryonic stem cells with a stromal cell line, such as a clonal pre-adipocyte stromal cell line PA6 provided the highest efficient derivation of DA neurons (Song et al. 2008; Guloglu and Larsen 2016). Up to 80% of cells expressed the specific cell marker TH, which is the rate-limiting enzyme in the synthesis of DA (Guloglu et al. 2014). However, the mechanism of differentiation is unclear, and considering that clinical cell-replacement therapy is a goal of stem cell research, exclusive reaction, and contamination risks may arise from the above method when in contact with animal products.

In this study, we tried to use NSC to achieve cell-replacement therapy. According to the experimental results, we proved that NSC could directly differentiate into TH^+^ neurons in a neuron differentiation medium. Although the intrinsic control of dopaminergic fate specification remains to be clarified, a higher percentage of the NSC could differentiate into TH^+^ cells after adding SAF. By using flow cytometry and real time PCR, We observed that TH and DAT were up-regulated in the SAF group and most of cells expressed TH and DA. Furthermore, the ELISA assay also suggested TH^+^ cells were able to synthesis and release more DA after treatment with SAF. So far, there are several reports on the transplantation of stem cell-derived cells into PD models, but the cell survival rate is low (Love et al. 2002; Ben-Hur et al. 2004; Kriks et al. 2011). We applied intrastriatal transplantation of PD rats to test the function of our NSC *in vivo*. Two weeks after implantation, we found that differentiated cells survived in the brain and the number of transplanted cells was much more in the SAF group.TH positive cells presenting in the brain were also much more, indicating that SAF promoted the differentiation and survival of DA cells. However, more work should be done to achieve survival, maintenance and function of NSC *in vivo*.

In conclusion, we used a simple culture system to efficiently generate TH^+^ cells from NSC. SAF promoted the production of functional DA cells *in vitro*. And these cells could survive and alleviate PD-induced death.
